# Supplementary material for: Potential causal association between leisure sedentary behaviors, physical activity and musculoskeletal health: A Mendelian randomization study
Source: PLoS One. 2023 Mar 16;18(3):e0283014. doi: 10.1371/journal.pone.0283014 (PMC10019723; doi:10.1371/journal.pone.0283014)
Supplement: S1 File — (DOC) [file pone.0283014.s001.doc]

**Supporting information**

**Potential causal association between leisure sedentary behaviors, physical activity and musculoskeletal health: a Mendelian randomization study**

Table S1. Instrument variables of television watching

Table S2. Instrument variables of computer use

Table S3. Instrument variables of moderate to vigorous physical activity

Table S4. Instrument variables of accelerometer-assessed physical activity

Table S5. MR estimates of the causal association between leisure sedentary behaviors, physical activity and musculoskeletal disorders

Table S6. Statistical power calculation for Mendelian randomization effects derived from inverse variance weighted method

Fig S1. Funnel plot for genetically predicted television watching on lower back pain

Fig S2. Funnel plot for genetically predicted television watching on intervertebral disc disorder

Fig S3. Funnel plot for genetically predicted computer use on intervertebral disc disorder

Fig S4. Funnel plot for genetically predicted computer use on rheumatoid arthritis

Fig S5. Forest plot (A) and leave-one-out analysis (B) for television watching on lower back pain

Fig S6. Forest plot (A) and leave-one-out analysis (B) for television watching on intervertebral disc disorder

Fig S7. Forest plot (A) and leave-one-out analysis (B) for computer use on intervertebral disc disorder

Fig S8. Forest plot (A) and leave-one-out analysis (B) for computer use on rheumatoid arthritis

| **Table S1. Instrument variables of television watching** | | | | | | |
| --- | --- | --- | --- | --- | --- | --- |
| SNP | effect_allele | other_allele | eaf | beta | se | pval |
| rs11810109 | A | T | 0.701438 | 0.016252 | 0.002357 | 5.40E-12 |
| rs984409 | G | A | 0.363032 | -0.01487 | 0.002253 | 4.10E-11 |
| rs17379561 | A | T | 0.855876 | -0.02548 | 0.003072 | 1.10E-16 |
| rs4845364 | A | G | 0.495003 | -0.0153 | 0.002156 | 1.30E-12 |
| rs6673341 | T | G | 0.465333 | -0.01451 | 0.002169 | 2.20E-11 |
| rs10737620 | T | A | 0.273668 | 0.014414 | 0.002419 | 2.60E-09 |
| rs6721975 | T | C | 0.231524 | -0.01669 | 0.002614 | 1.70E-10 |
| rs72781699 | G | A | 0.797367 | -0.01868 | 0.002678 | 3.00E-12 |
| rs12105701 | C | T | 0.395851 | -0.01288 | 0.002208 | 5.40E-09 |
| rs7564130 | T | C | 0.640601 | -0.01499 | 0.002251 | 2.80E-11 |
| rs10189857 | A | G | 0.56785 | -0.02046 | 0.00218 | 6.20E-21 |
| rs62641636 | A | G | 0.691839 | 0.014424 | 0.002339 | 7.00E-10 |
| rs11689199 | A | G | 0.597665 | 0.018464 | 0.002204 | 5.50E-17 |
| rs1451533 | G | A | 0.724835 | -0.0157 | 0.002433 | 1.10E-10 |
| rs374722 | G | A | 0.150476 | 0.024493 | 0.003024 | 5.50E-16 |
| rs3754970 | T | C | 0.495822 | -0.015 | 0.00217 | 4.80E-12 |
| rs4577309 | A | G | 0.468698 | 0.016004 | 0.002168 | 1.60E-13 |
| rs10932837 | C | T | 0.494411 | -0.01311 | 0.002162 | 1.30E-09 |
| rs4973576 | C | A | 0.298072 | -0.01452 | 0.002379 | 1.00E-09 |
| rs9834970 | T | C | 0.501031 | 0.012768 | 0.002158 | 3.30E-09 |
| rs3796386 | G | A | 0.571888 | -0.02617 | 0.00218 | 3.20E-33 |
| rs11130793 | C | T | 0.601737 | 0.012922 | 0.002211 | 5.00E-09 |
| rs11714337 | G | A | 0.569041 | 0.014402 | 0.002189 | 4.70E-11 |
| rs6797840 | A | C | 0.455899 | -0.0161 | 0.002184 | 1.70E-13 |
| rs2034768 | A | G | 0.48743 | 0.014736 | 0.002158 | 8.60E-12 |
| rs9867121 | C | A | 0.817728 | 0.019499 | 0.002809 | 3.90E-12 |
| rs12491503 | G | A | 0.670306 | -0.01426 | 0.002299 | 5.50E-10 |
| rs114600294 | G | C | 0.788982 | -0.01624 | 0.002642 | 7.90E-10 |
| rs66852340 | C | T | 0.775527 | -0.01779 | 0.002601 | 7.90E-12 |
| rs34811474 | G | A | 0.767813 | 0.015283 | 0.002557 | 2.30E-09 |
| rs6850494 | A | C | 0.615571 | -0.01433 | 0.002222 | 1.10E-10 |
| rs13107325 | C | T | 0.925644 | -0.02917 | 0.004123 | 1.50E-12 |
| rs7693703 | G | A | 0.910033 | 0.022844 | 0.003841 | 2.70E-09 |
| rs6825241 | C | A | 0.536204 | -0.01696 | 0.002166 | 4.90E-15 |
| rs7693082 | G | C | 0.299686 | 0.015072 | 0.002358 | 1.70E-10 |
| rs262890 | A | G | 0.698905 | -0.01857 | 0.002356 | 3.20E-15 |
| rs7716447 | A | G | 0.639406 | -0.01338 | 0.002269 | 3.60E-09 |
| rs1031423 | T | C | 0.215208 | -0.01852 | 0.002626 | 1.80E-12 |
| rs57585211 | T | G | 0.826832 | -0.01673 | 0.00285 | 4.30E-09 |
| rs10041724 | T | C | 0.807695 | 0.018093 | 0.002739 | 3.90E-11 |
| rs62379379 | G | T | 0.928551 | -0.02602 | 0.004232 | 7.80E-10 |
| rs10054327 | G | A | 0.576024 | 0.017245 | 0.00219 | 3.40E-15 |
| rs42210 | G | C | 0.289169 | -0.01385 | 0.002395 | 7.30E-09 |
| rs72828890 | C | T | 0.869256 | 0.019292 | 0.003298 | 4.90E-09 |
| rs72834698 | G | A | 0.858074 | 0.022656 | 0.003101 | 2.70E-13 |
| rs9471333 | C | T | 0.450187 | 0.013104 | 0.002169 | 1.50E-09 |
| rs6905544 | A | G | 0.398622 | -0.01899 | 0.00221 | 8.50E-18 |
| rs17789218 | T | C | 0.75605 | 0.018597 | 0.002514 | 1.40E-13 |
| rs2184364 | A | G | 0.782382 | 0.01564 | 0.002637 | 3.00E-09 |
| rs9718104 | T | G | 0.94155 | -0.04077 | 0.00461 | 9.30E-19 |
| rs17568389 | T | A | 0.486903 | 0.015481 | 0.002165 | 8.60E-13 |
| rs62471080 | G | C | 0.540782 | -0.01278 | 0.002167 | 3.70E-09 |
| rs7834121 | G | T | 0.504369 | -0.01396 | 0.002164 | 1.10E-10 |
| rs6472942 | T | C | 0.568486 | -0.01316 | 0.002188 | 1.80E-09 |
| rs2616830 | G | A | 0.461988 | 0.016465 | 0.002166 | 2.90E-14 |
| rs34864022 | A | G | 0.933558 | -0.02638 | 0.004357 | 1.40E-09 |
| rs12554512 | T | C | 0.584071 | 0.020702 | 0.002193 | 3.80E-21 |
| rs870151 | T | A | 0.527632 | -0.01563 | 0.002187 | 8.80E-13 |
| rs7043521 | A | T | 0.430653 | 0.01427 | 0.002185 | 6.50E-11 |
| rs4382592 | T | G | 0.301425 | 0.013672 | 0.002358 | 6.70E-09 |
| rs2073869 | C | T | 0.833626 | 0.018611 | 0.002908 | 1.50E-10 |
| rs1243182 | C | T | 0.689639 | -0.01859 | 0.002342 | 2.00E-15 |
| rs2045147 | A | G | 0.449407 | 0.012669 | 0.002177 | 5.90E-09 |
| rs10786658 | A | T | 0.413503 | -0.01445 | 0.002196 | 4.60E-11 |
| rs11245482 | T | C | 0.61325 | -0.01323 | 0.002221 | 2.60E-09 |
| rs17727474 | C | T | 0.832295 | 0.017551 | 0.002961 | 3.10E-09 |
| rs801733 | A | C | 0.641215 | 0.016859 | 0.002253 | 7.30E-14 |
| rs17207890 | G | A | 0.657407 | 0.015694 | 0.002287 | 6.70E-12 |
| rs11218575 | C | T | 0.566037 | 0.015375 | 0.002187 | 2.10E-12 |
| rs10772643 | C | T | 0.107916 | 0.0248 | 0.003497 | 1.30E-12 |
| rs10771746 | C | T | 0.716605 | -0.01428 | 0.002395 | 2.50E-09 |
| rs10876864 | G | A | 0.427197 | -0.01334 | 0.002184 | 1.00E-09 |
| rs8756 | C | A | 0.484619 | -0.01345 | 0.002166 | 5.30E-10 |
| rs2173650 | G | T | 0.852206 | 0.017845 | 0.003044 | 4.60E-09 |
| rs9563168 | G | A | 0.791482 | 0.017587 | 0.002667 | 4.30E-11 |
| rs9569734 | A | G | 0.843521 | 0.018885 | 0.003007 | 3.40E-10 |
| rs56858768 | G | A | 0.700762 | -0.01488 | 0.00237 | 3.40E-10 |
| rs7991062 | C | G | 0.658663 | -0.01774 | 0.002287 | 8.80E-15 |
| rs10145592 | C | G | 0.409418 | -0.01485 | 0.002208 | 1.80E-11 |
| rs2460 | G | A | 0.736281 | -0.01529 | 0.002459 | 5.00E-10 |
| rs61331678 | G | C | 0.567932 | 0.015046 | 0.002187 | 6.10E-12 |
| rs7189927 | T | C | 0.355786 | 0.014974 | 0.002259 | 3.40E-11 |
| rs749671 | G | A | 0.62863 | 0.015654 | 0.002238 | 2.70E-12 |
| rs7184800 | G | A | 0.696784 | 0.016817 | 0.002349 | 8.20E-13 |
| rs142710267 | T | G | 0.65012 | 0.016245 | 0.002366 | 6.60E-12 |
| rs2447098 | C | A | 0.475175 | -0.01495 | 0.002178 | 6.80E-12 |
| rs2584597 | T | C | 0.662057 | 0.0151 | 0.002396 | 2.90E-10 |
| rs9902312 | T | C | 0.683148 | 0.015324 | 0.002327 | 4.50E-11 |
| rs303753 | G | A | 0.652948 | -0.01446 | 0.002291 | 2.70E-10 |
| rs9964724 | C | T | 0.317881 | 0.017647 | 0.002327 | 3.30E-14 |
| rs7248205 | C | T | 0.397614 | 0.013925 | 0.002217 | 3.40E-10 |
| rs111901094 | G | T | 0.817212 | -0.01708 | 0.002847 | 2.00E-09 |
| rs6131281 | C | T | 0.596509 | 0.016081 | 0.002206 | 3.10E-13 |
| rs6141814 | C | A | 0.613455 | -0.0135 | 0.002226 | 1.30E-09 |
| rs56103247 | C | T | 0.943942 | 0.029765 | 0.004752 | 3.80E-10 |

| **Table S2. Instrument variables of computer use** | | | | | | |
| --- | --- | --- | --- | --- | --- | --- |
| SNP | effect_allele | other_allele | eaf | beta | se | pval |
| rs2748985 | T | C | 0.454667 | -0.01534 | 0.002213 | 4.10E-12 |
| rs6744254 | C | T | 0.47126 | -0.0159 | 0.002204 | 5.40E-13 |
| rs10208088 | C | T | 0.418634 | 0.013103 | 0.002232 | 4.40E-09 |
| rs11708955 | T | C | 0.694765 | -0.01575 | 0.002387 | 4.20E-11 |
| rs6774533 | C | T | 0.294245 | -0.01494 | 0.002458 | 1.20E-09 |
| rs2068625 | T | C | 0.302374 | -0.01584 | 0.002398 | 4.00E-11 |
| rs2220599 | C | G | 0.632986 | -0.01605 | 0.00229 | 2.40E-12 |
| rs11749912 | A | G | 0.422554 | 0.01392 | 0.002237 | 4.90E-10 |
| rs9372625 | G | A | 0.61853 | -0.01839 | 0.002272 | 5.70E-16 |
| rs55772938 | A | G | 0.704749 | -0.01512 | 0.002417 | 3.90E-10 |
| rs2345941 | A | G | 0.550313 | 0.014647 | 0.002217 | 4.00E-11 |
| rs13262595 | A | G | 0.440345 | -0.01573 | 0.002215 | 1.20E-12 |
| rs4977839 | G | A | 0.584465 | -0.01992 | 0.002233 | 4.70E-19 |
| rs113851275 | G | A | 0.891576 | -0.02094 | 0.003537 | 3.20E-09 |
| rs73578186 | C | T | 0.675347 | 0.01499 | 0.002365 | 2.30E-10 |
| rs2734849 | A | G | 0.491453 | -0.01351 | 0.0022 | 8.20E-10 |
| rs1448355 | C | T | 0.381282 | -0.01461 | 0.002274 | 1.30E-10 |
| rs35933007 | G | A | 0.771615 | -0.01519 | 0.002644 | 9.20E-09 |
| rs206965 | T | C | 0.206729 | 0.015601 | 0.002715 | 9.10E-09 |
| rs166835 | C | T | 0.443342 | 0.013136 | 0.002222 | 3.40E-09 |
| rs4073003 | A | G | 0.873541 | 0.020163 | 0.00332 | 1.20E-09 |
| rs984409 | A | T | 0.358909 | -0.01409 | 0.002303 | 9.30E-10 |
| rs631130 | A | G | 0.435739 | 0.013109 | 0.002227 | 4.00E-09 |

**Table S3. Instrument variables of moderate to vigorous physical activity**

| SNP | effect_allele | other_allele | beta | se | eaf | pval |
| --- | --- | --- | --- | --- | --- | --- |
| rs2942127 | A | G | -0.01604 | 0.00290278 | 0.824644 | 3.30E-08 |
| rs1974771 | A | G | 0.021339 | 0.00367836 | 0.099975 | 6.60E-09 |
| rs2114286 | G | A | 0.012245 | 0.00221725 | 0.534243 | 3.30E-08 |
| rs877483 | C | T | -0.01223 | 0.00222756 | 0.566815 | 4.00E-08 |
| rs2035562 | G | A | 0.013876 | 0.00235606 | 0.672483 | 3.90E-09 |
| rs1972763 | T | C | -0.01284 | 0.00232366 | 0.657628 | 3.30E-08 |
| rs77742115 | C | T | 0.018348 | 0.00319777 | 0.138319 | 9.60E-09 |
| rs2854277 | T | C | -0.03203 | 0.00506676 | 0.082571 | 2.60E-10 |
| rs7804463 | C | T | -0.01501 | 0.00221333 | 0.470424 | 1.20E-11 |
| rs921915 | C | T | 0.013888 | 0.00224013 | 0.587905 | 5.70E-10 |
| rs1186721 | A | G | 0.01299 | 0.00237226 | 0.315844 | 4.40E-08 |
| rs1043595 | A | G | -0.01441 | 0.00245416 | 0.282865 | 4.30E-09 |
| rs2988004 | G | T | 0.013171 | 0.00223979 | 0.442245 | 4.10E-09 |
| rs7326482 | T | G | 0.012961 | 0.00229416 | 0.615163 | 1.60E-08 |
| rs10145335 | A | G | 0.014122 | 0.00254139 | 0.250611 | 2.70E-08 |
| rs12912808 | T | C | -0.01755 | 0.00310889 | 0.148607 | 1.70E-08 |
| rs4886868 | G | T | 0.012495 | 0.00226611 | 0.585862 | 3.50E-08 |
| rs429358 | C | T | 0.021982 | 0.00305356 | 0.154172 | 6.10E-13 |
| rs1921981 | A | G | -0.01304 | 0.00237139 | 0.325647 | 3.80E-08 |

| **Table S4. Instrument variables of accelerometer-assessed physical activity** | | | | | | |
| --- | --- | --- | --- | --- | --- | --- |
| SNP | effect_allele | other_allele | eaf | beta | se | pval |
| rs34517439 | A | C | 0.120938 | -0.30792 | 0.0562443 | 4.40E-08 |
| rs6775319 | T | A | 0.729248 | -0.225081 | 0.040802 | 3.50E-08 |
| rs9293503 | C | T | 0.111505 | -0.328953 | 0.0586852 | 2.10E-08 |
| rs12522261 | A | G | 0.343473 | -0.210522 | 0.0383038 | 3.90E-08 |
| rs11012732 | G | A | 0.332193 | -0.224986 | 0.0385641 | 5.40E-09 |
| rs148193266 | C | A | 0.042668 | 0.510362 | 0.0921544 | 3.10E-08 |
| rs56194509 | G | T | 0.220486 | 0.303404 | 0.0439408 | 5.00E-12 |
| rs59499656 | T | A | 0.344493 | 0.228253 | 0.0382563 | 2.40E-09 |

**Table S5. MR estimates of the causal association between leisure sedentary behaviors, physical activity and musculoskeletal disorders**

| **Exposure** | **Methods** | **Lower back pain** | | **Intervertebral disc disorder** | | **Bone mineral density** | | **Rheumatoid arthritis** | |
| --- | --- | --- | --- | --- | --- | --- | --- | --- | --- |
|  |  | OR (95% CI) | *P* | OR (95% CI) | *P* | β (95% CI) | *P* | OR (95% CI) | *P* |
| MVPA | IVW | 0.78(0.47,1.27) | 0.319 | 0.97(0.60,1.56) | 0.885 | -0.02(-0.31, 0.26) | 0.868 | 1.06(0.47, 2.41) | 0.89 |
|  | MR-Egger | 0.13(0.01,1.95) | 0.158 | 0.39(0.02,6.25) | 0.516 | 0.53(-0.76, 1.82) | 0.435 | 8.38(0.08, 914.51) | 0.39 |
|  | Weighted median | 0.8(0.47, 1.37) | 0.422 | 0.85(0.52,1.39) | 0.517 | 0.045(-0.23, 0.32) | 0.750 | 2.08(0.76, 5.72) | 0.15 |
| APA | IVW | 0.94(0.87,1.01) | 0.070 | 0.99(0.94,1.04) | 0.634 | 0.01(-0.02, 0.04) | 0.502 | 0.98(0.86, 1.12) | 0.78 |
|  | MR-Egger | 1.1(0.81, 1.51) | 0.552 | 1.20(1.00,1.44) | 0.102 | -0.004(-0.15, 0.15) | 0.962 | 0.65(0.29, 1.43) | 0.36 |
|  | Weighted median | 0.94(0.89,0.99) | 0.015 | 0.98(0.93,1.02) | 0.332 | 0.007(-0.02, 0.04) | 0.619 | 0.98(0.88, 1.09) | 0.69 |
| Television watching | IVW | 1.68(1.41,2.01) | 8.23E-09 | 1.62(1.37,1.91) | 2.13E-08 | -0.07(0.16, 0.02) | 0.138 | 2.28(1.56, 3.33) | 2.25E-05 |
|  | MR-Egger | 2.31(1.00,5.34) | 0.055 | 1.67(0.75,3.74) | 0.213 | 0.05(-0.40, 0.50) | 0.822 | 0.60(0.10, 3.58) | 0.58 |
|  | Weighted median | 1.54(1.21,1.95) | 0.0004 | 1.56(1.27,1.93) | 3.19E-05 | -0.10(-0.22, 0.02) | 0.115 | 2.18(1.36, 3.48) | 0.001 |
| Computer use | IVW | 0.48(0.35,0.66) | 8.54E-06 | 0.6(0.42, 0.86) | 0.005 | -0.09(-0.37, 0.20) | 0.552 | 0.28(0.13, 0.60) | 0.001 |
|  | MR-Egger | 1.02(0.08,13.39) | 0.987 | 0.43(0.03,6.80) | 0.554 | 0.49(-1.86, 2.83) | 0.688 | 0.01(0.00001,6.73) | 0.18 |
|  | Weighted median | 0.51(0.32,0.81) | 0.004 | 0.73(0.49,1.09) | 0.126 | -0.18(-0.45, 0.10) | 0.206 | 0.26(0.10, 0.67) | 0.006 |

MR, Mendelian randomization; IVW, inverse variance weighted; PA, physical activity; MVPA, moderate to vigorous physical activity; APA, Accelerometer assessed PA.

**Table S6. Statistical power calculation for Mendelian randomization effects derived from inverse variance weighted method**

| Exposures | Outcomes | R2 | Power (%) |
| --- | --- | --- | --- |
| Television watching | Lower back pain | 0.0105 | 100 |
| Television watching | Intervertebral disc disorder | 0.0105 | 100 |
| Computer use | Intervertebral disc disorder | 0.0024 | 92 |
| Computer use | Rheumatoid arthritis | 0.0024 | 100 |


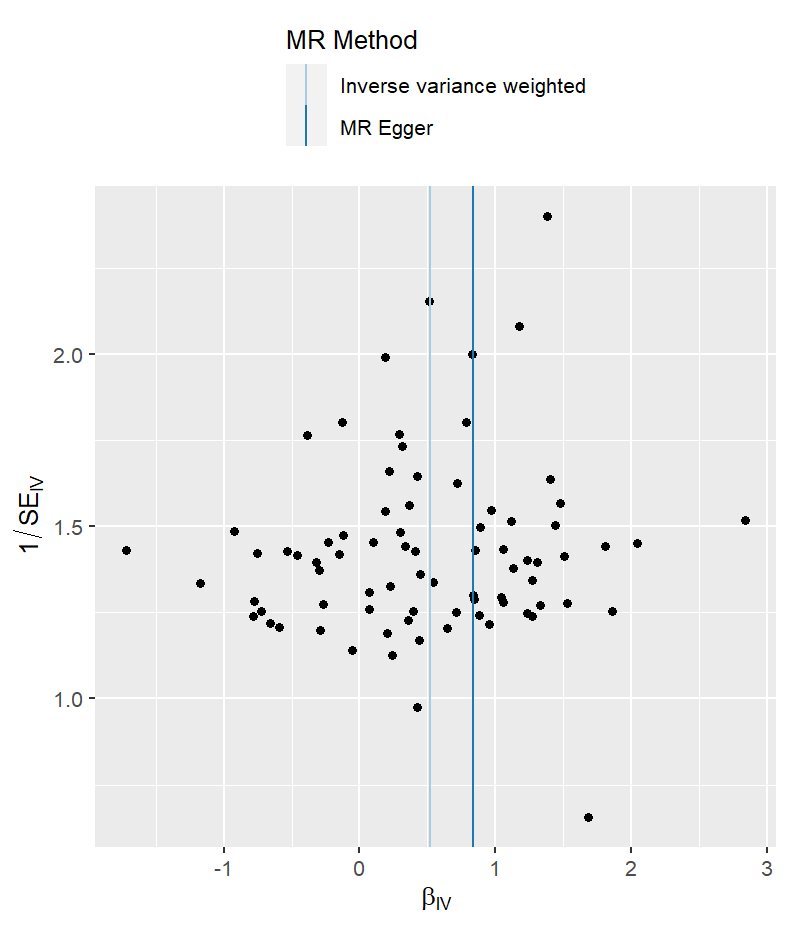


**Fig S1. Funnel plot for genetically predicted television watching on lower back pain**


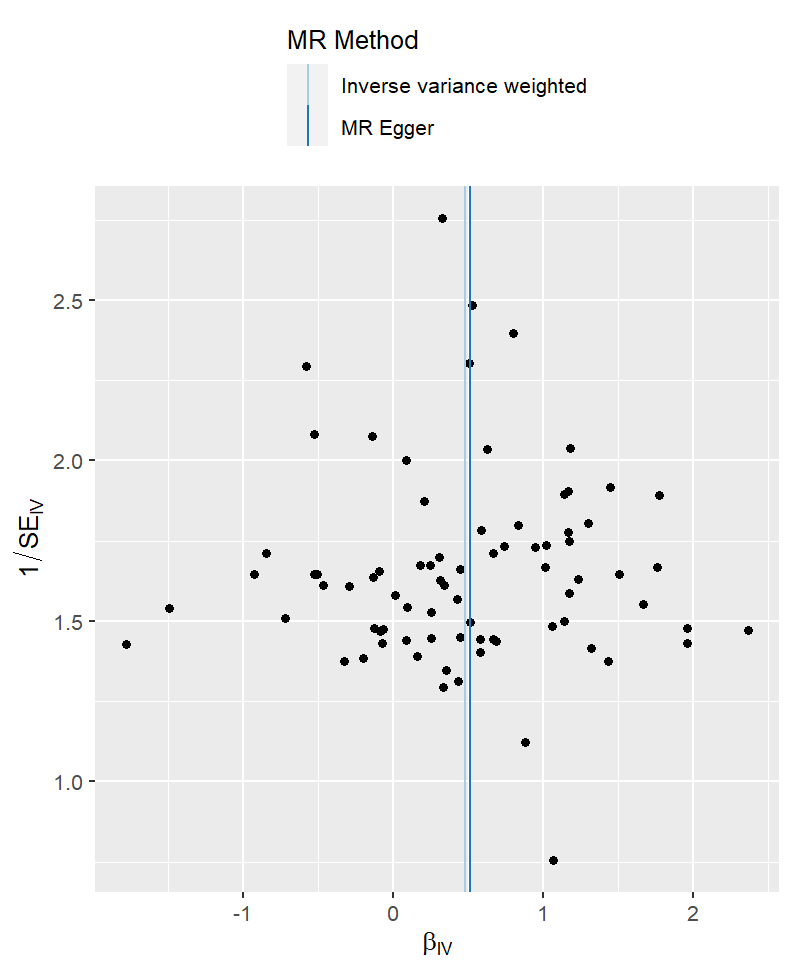


**Fig S2. Funnel plot for genetically predicted television watching on intervertebral disc disorder**


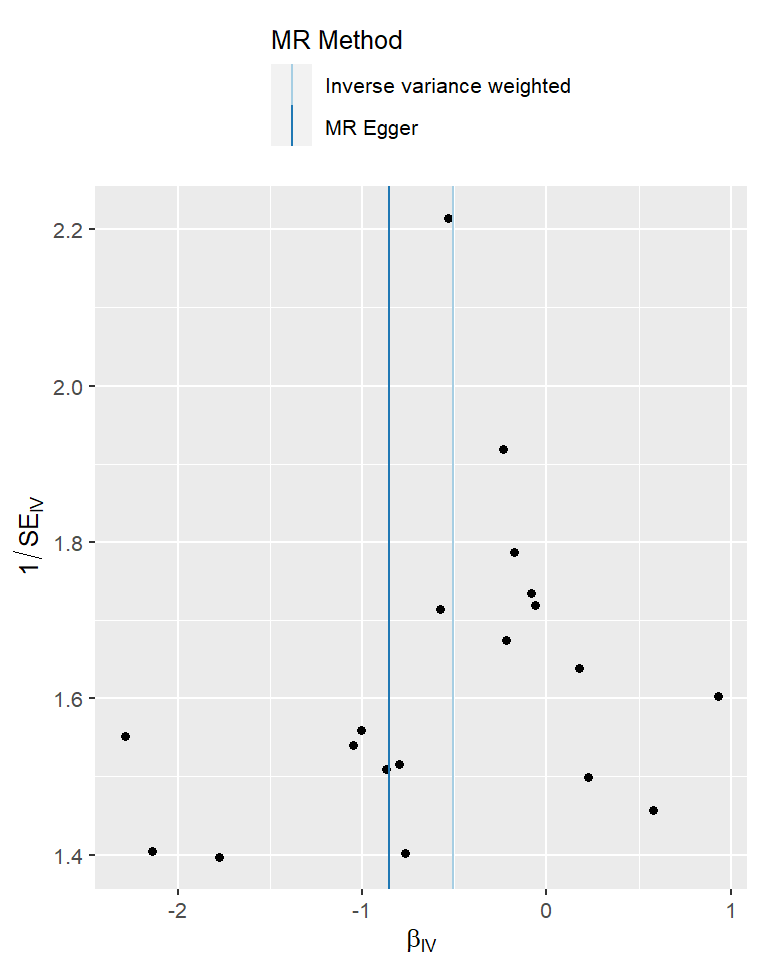


**Fig S3. Funnel plot for genetically predicted computer use on intervertebral disc disorder**


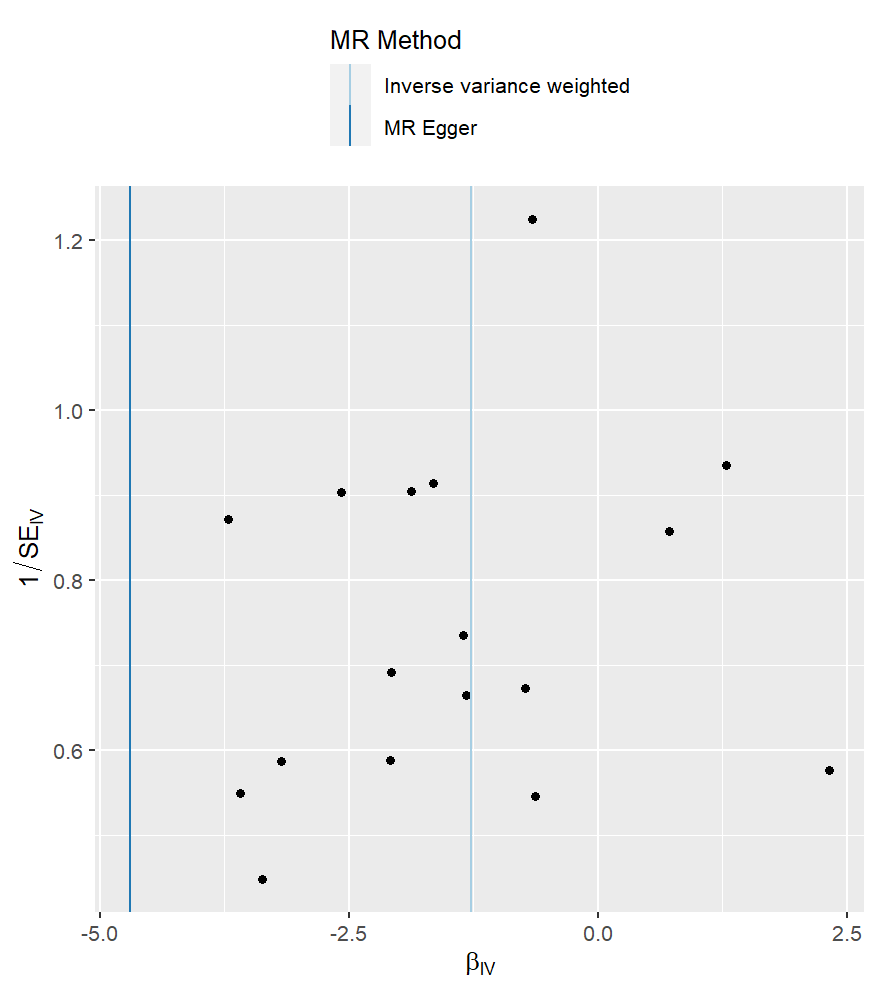


**Fig S4. Funnel plot for genetically predicted computer use on rheumatoid arthritis**


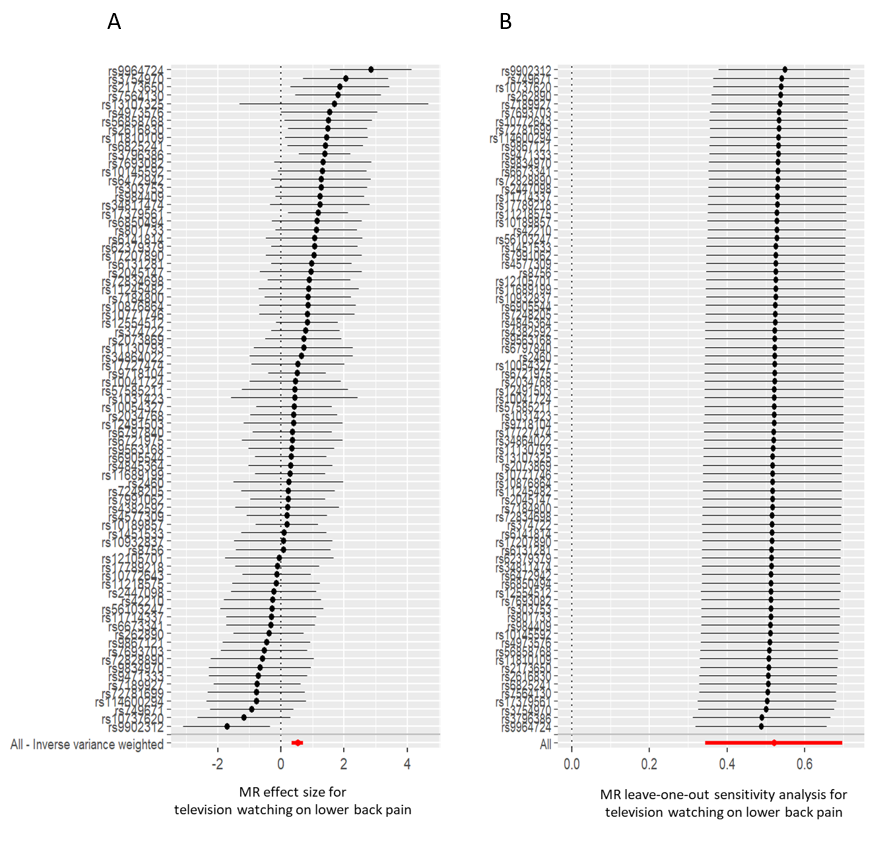


**Fig S5. Forest plot (A) and leave-one-out analysis (B) for television watching on lower back pain**.


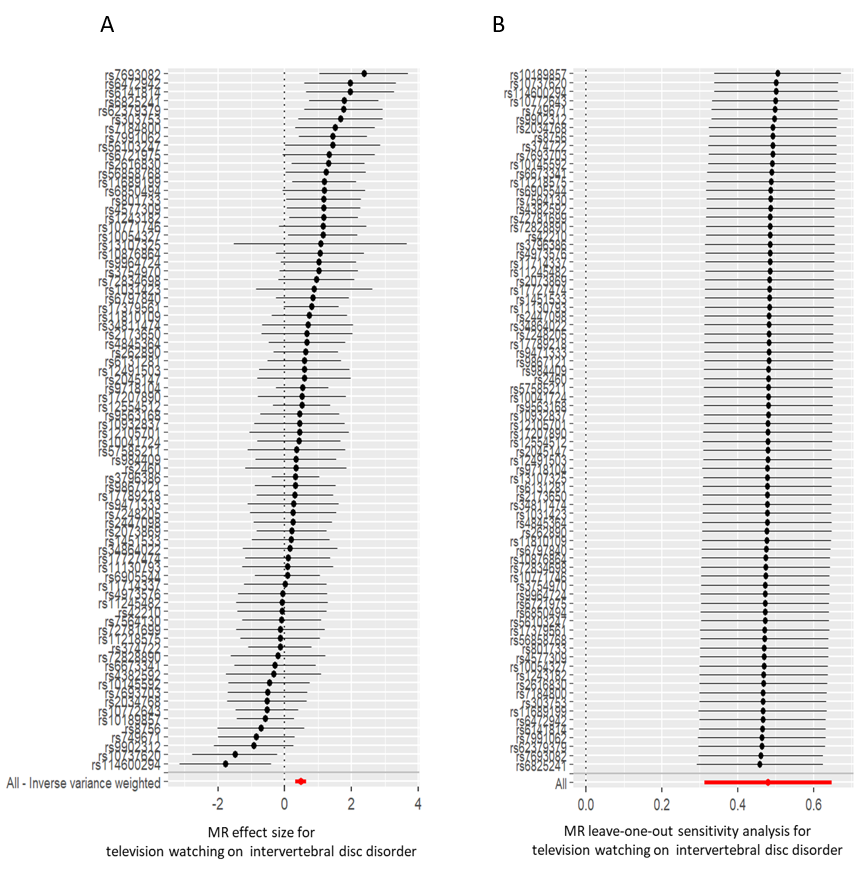


**Fig S6. Forest plot (A) and leave-one-out analysis (B) for television watching on intervertebral disc disorder.**


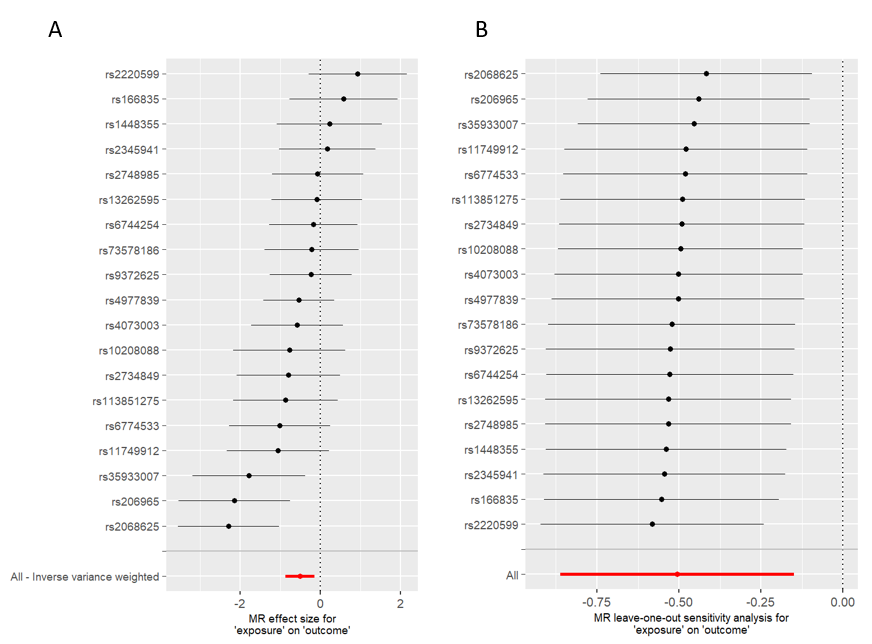


**Fig S7. Forest plot (A) and leave-one-out analysis (B) for computer use on intervertebral disc disorder**

**
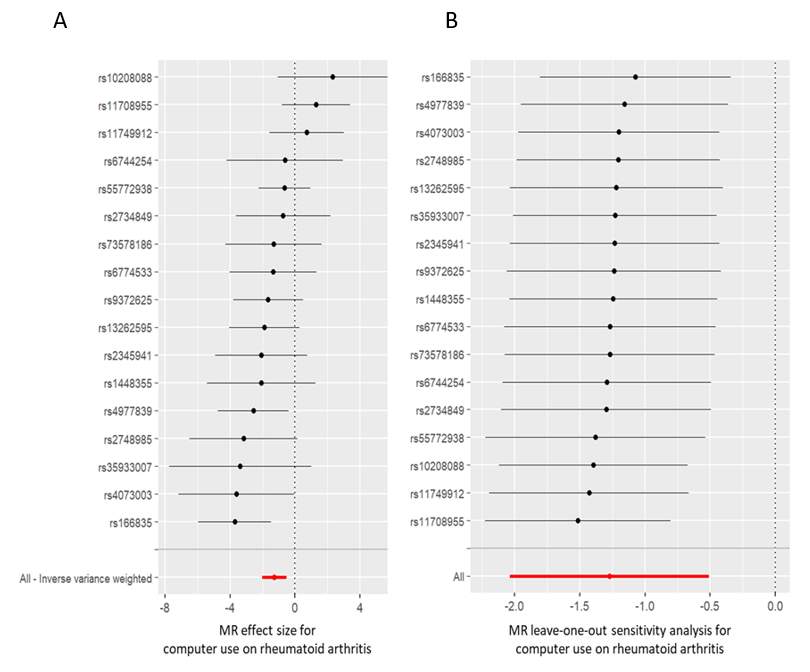
**

**Fig S8. Forest plot (A) and leave-one-out analysis (B) for computer use on rheumatoid arthritis.**
