# Supplementary material for: Potential causal association between leisure sedentary behaviors, physical activity and musculoskeletal health: A Mendelian randomization study
Source: PLoS One. 2023 Mar 16;18(3):e0283014. doi: 10.1371/journal.pone.0283014 (PMC10019723; doi:10.1371/journal.pone.0283014)
Supplement: Attachment — Submitted filename: Response to Reviewers.docx [file pone.0283014.s002.docx]

We appreciate the editors and reviewers very much for their comments and suggestions on our manuscript entitled“Potential causal association between leisure sedentary behaviors, physical activity and musculoskeletal health: a Mendelian randomization study”. We have made revisions which are marked in red in the article. The revised manuscript has been uploaded.

Journal Requirements:

When submitting your revision, we need you to address these additional requirements.

1. Please ensure that your manuscript meets PLOS ONE's style requirements, including those for file naming. The PLOS ONE style templates can be found at

https://journals.plos.org/plosone/s/file?id=wjVg/PLOSOne_formatting_sample_main_body.pdf and https://journals.plos.org/plosone/s/file?id=ba62/PLOSOne_formatting_sample_title_authors_affiliations.pdf

We revise the article according to PLOS ONE's style.

2. We note that the grant information you provided in the ‘Funding Information’ and ‘Financial Disclosure’ sections do not match.

When you resubmit, please ensure that you provide the correct grant numbers for the awards you received for your study in the ‘Funding Information’ section.

We ensure that we provide the correct ‘Funding Information’.

3. Thank you for stating in your Funding Statement:

"This project was supported by the National Natural Science Foundation of China (8200153156) and Xinglin Scholars Project of Chengdu University of Traditional Chinese Medicine (BSH2020009)."

Please provide an amended statement that declares *all* the funding or sources of support (whether external or internal to your organization) received during this study, as detailed online in our guide for authors at http://journals.plos.org/plosone/s/submit-now. Please also include the statement “There was no additional external funding received for this study.” in your updated Funding Statement.

Please include your amended Funding Statement within your cover letter. We will change the online submission form on your behalf.

We have added the statement “There was no additional external funding received for this study.”

4. Thank you for stating the following financial disclosure:

"This project was supported by the National Natural Science Foundation of China (8200153156) and Xinglin Scholars Project of Chengdu University of Traditional Chinese Medicine (BSH2020009). The funders had no role in study design, data collection and analysis, decision to publish, or preparation of the manuscript."

We note that one or more of the authors is affiliated with the funding organization, indicating the funder may have had some role in the design, data collection, analysis or preparation of your manuscript for publication; in other words, the funder played an indirect role through the participation of the co-authors. If the funding organization did not play a role in the study design, data collection and analysis, decision to publish, or preparation of the manuscript and only provided financial support in the form of authors' salaries and/or research materials, please do the following:

a. Review your statements relating to the author contributions, and ensure you have specifically and accurately indicated the role(s) that these authors had in your study. These amendments should be made in the online form.

b. Confirm in your cover letter that you agree with the following statement, and we will change the online submission form on your behalf:

“The funder provided support in the form of salaries for authors [insert relevant initials], but did not have any additional role in the study design, data collection and analysis, decision to publish, or preparation of the manuscript. The specific roles of these authors are articulated in the ‘author contributions’ section.”"

"This project was supported by the National Natural Science Foundation of China (8200153156) and Xinglin Scholars Project of Chengdu University of Traditional Chinese Medicine (BSH2020009). The funders had no role in study design, data collection and analysis, decision to publish, or preparation of the manuscript."

5. PLOS requires an ORCID iD for the corresponding author in Editorial Manager on papers submitted after December 6th, 2016. Please ensure that you have an ORCID iD and that it is validated in Editorial Manager. To do this, go to ‘Update my Information’ (in the upper left-hand corner of the main menu), and click on the Fetch/Validate link next to the ORCID field. This will take you to the ORCID site and allow you to create a new iD or authenticate a pre-existing iD in Editorial Manager. Please see the following video for instructions on linking an ORCID iD to your Editorial Manager account: https://www.youtube.com/watch?v=_xcclfuvtxQ

We added the ORCID iD for the corresponding author.

5. Review Comments to the Author

Please use the space provided to explain your answers to the questions above. You may also include additional comments for the author, including concerns about dual publication, research ethics, or publication ethics. (Please upload your review as an attachment if it exceeds 20,000 characters)

**Reviewer #1:** Summary Paragraph

The authors present a Mendelian Randomization study assessing the potential causal associations between leisure sedentary behaviors and physical activity phenotypes and a variety of musculoskeletal disorders such as lower-back pain, intervertebral disc disorder, rheumatoid arthritis, and bone mineral density. This work has the potential of increasing the understanding of the potential causal relationships between known risk factors for musculoskeletal disorders and this outcome using genetically based instrumental variables. One of my main concerns is the use of strong causal language in the title, discussion section and conclusions. Another concern is the reduced scope of interpretations of these results in the discussion (more extensive explanations about biological mechanism [e.g., from SNPs to tissues], and about potential clinical relevance would be needed).

Strengths:

The authors explained well the background of the study and justification for using MR. Exposure and outcome variables are clear in the introduction and throughout the manuscript. The authors outlined well how the exposure and outcome variables have a plausible relationship. The assumptions and the study design were described clearly and tested, quality control and selection of genetic instruments were also well outlined.

Weaknesses or areas for improvement:

Since biological mechanisms, their interaction across time, type and time of measurements and MR assumptions play a crucial role here, the use of strong causal language in the title, discussion section and conclusions could undermine the words of caution when interpreting results from MR analyses.

We have revised the article according to the suggestion.

Points for clarification are outlined below by section.

Title:

• I’d suggest the authors to add the word “Potential” before “Causal association (…)” in the title.

We added the word “Potential” before “Causal association (…)” in the title.

Abstract:

• (p. 8) Usually MR hypotheses include causal language or language inferring the potential causality. In the abstract’s background, the authors use “related”, which hints more to non-directional associations in their hypothesis.

We revised the article.

Introduction:

• (p. 9) I'd suggest to clarify "It" after the reference call #7. Although it can be assumed what the authors are talking about, it is usually best to specify the subject of the new sentence, especially after a long multi-clause one.

We have used the word " Osteoporosis " instead of "It".

• (p. 9) Add a few examples of the unique benefits of exercise for the musculoskeletal system.

We have added a few examples of the unique benefits of exercise.

Method:

• (p. 10) Although it might be assumed that the participants for the two phenotypes of PA have European ancestry, specify if this is the case in the corresponding paragraph under the 2.2 subsection.

The participants for the two phenotypes of PA have European ancestry. We added this to the article.

• (p. 11) Add an “Ancestry” column in Table 1 if multiple ancestries were used. A column listing the data sources for each Consortium could also be helpful for Table 1 (or this list could also be in the notes part of Table 1 to save space).

We have added an “Ancestry” column in Table 1.

• (p. 12) It would be interesting to inspect the differences of significance from unadjusted p-values to at least two multiple-testing adjustments. The authors included the Bonferroni adjustment, which is conservative and may increase Type-II error. Would the authors be able to estimate p-values under a less conservative method (e.g., false discovery rate adjustment) and add them next to the Bonferroni adjusted ones?

To account for multiple testing in our analyses, a Bonferroni-corrected P-value was set as 0.05/16 (0.003). P < 0.003 from MR analysis was regarded as statistically significant, and P < 0.05 was considered nominally significant.

• Were any power calculations performed prior to the analysis? Whether these calculations were performed or not, it would be interesting to see the differences in statistical power from each sample.

We calculated the statistic power for important estimates. A power of over 90% was observed. Statistical power was presented in Table S6.

• Are the authors able to provide more information about the covariates beyond BDM? And mention whether these covariates were adjusted for across samples or not?

Different GWAS data may be adjusted for different covariates. However, to obtain unbiased causal effects, genetic instrumental variables were selected by the rigorous criteria.

• Would the authors be able to provide information about the characteristics of missing data? And if present, how the missingness was addressed?

Genetic instrumental variables were selected after rigorous criteria. For example, SNPs with F-statistic less than 10 were removed.

• Were this study and its protocols pre-registered? If so, where can the reader find them?

The GWAS data included in this paper are all publicly available. This study was not pre-registered.

Results:

• Since this study used a two-sample MR approach, are the authors able to include information about the variant-exposure correlations between the samples of exposure and outcome phenotypes? And, would the authors also be able to include any report of participant overlap between exposure and outcome samples?

GWAS data about exposures are all from the UK Biobank，but GWAS data about outcomes were obtained from the FinnGen consortium and meta-analysis of GWAS. Therefore, there is very little sample overlap and it did not invalidate the MR results.

• Did the authors test the direction of causation in the opposite direction (i.e., outcome -> exposure, or bidirectional MR)? Many biological mechanisms are complex and might include feedback loops that may hint scientist to test the plausibility of the reverse direction of causation (e.g., causal influences of outcome on exposure at unmeasured timepoints). If the authors tested this direction, would they be able to provide the results? If they didn’t test it, would they be able add this supplementary analysis to test this direction?

Considering that LBS and PA belong to behaviors instead of diseases, it may not be appropriate to use them as outcome phenotypes. Therefore, we did not perform a bidirectional MR.

Discussion:

• Would the authors be able to discuss any potential clinical relevance and public health applications of these results beyond the discussion provided?

We revised the article according to the suggestion.

• The authors mentioned in the limitations paragraph that these results cannot be generalized to populations of other ancestries different than European, which is true. Are the authors able to discuss the importance of expanding the genetic and phenotypic research more to non-European ancestries? If so, would they inform the reader about their effort to find datasets/summary-stats of non-European ancestry for their analyses in this manuscript?

To reduce population-stratification bias, we extracted summarized data of European ancestry. This is a strength of our study. Meanwhile, it limits the generalizability of our findings to other ethnic groups. At present, no aggregated data from genome-wide association studies of non-European ancestry are available for this MR study.

• (p. 15-16) I’d suggest to add “potential” before “causal relationship” in the following sentence “(…), the MR study revealed the causal relationship between LSB/PA and different musculoskeletal disorders (…)” and also in the many occurrences of “causal relationship” or “causal association” or other similar assertions throughout the discussion section and also importantly, in the Conclusions. It is always important to use causal language cautiously in MR studies.

We revised the article according to the suggestion.

Figures:

• This might be an issue of the pdf convertor, but I’d encourage the authors to double check that figures (and any image-based object/file) do not pixelate. Otherwise reading small letters or visualizing shapes can become difficult. I suggest to use vector-based images.

We checked these figures and resized the figures.

Other:

• Is there a link where readers can find the code used for the analyses? This can enhance transparency and reproducibility.

Data source and the link are shown in the article.

**Reviewer #2**: 1. The GWAS’s used have all been on individuals with European ancestry. I would request the authors to replicate these findings or at least perform these analyses in GWAS’s of other ancestries.

To reduce population-stratification bias, we extracted summarized data of European ancestry. This is a strength of our study. Meanwhile, it limits the generalizability of our findings to other ethnic groups. At present, no aggregated data from genome-wide association studies of other ancestries are available to conduct the MR study. Further studies will be done if data are available for other ancestries.

2. I would like the authors to perform the bi-directional MR also i.e. from musculoskeletal disorders to LSB/PA

Considering that LBS and PA belong to behaviors instead of diseases, it may not be appropriate to use them as outcome phenotypes. Therefore, we did not perform a bidirectional MR.

3. It is not clear to me why did the authors select RA, LBP, IVDD for musculoskeletal disorders? Were they just examples? Why did the authors not select carpal tunnel syndrome and tendonitis?

First, we selected several common musculoskeletal disorders. This study shows the association between LBS/PA and RA, LBP, IVDD, and BMD, but it cannot describe the association of LBS/PA with other musculoskeletal disorders. Second, observational studies have suggested that LBS/PA are associated with RA, LBP, IVDD, and BMD, so we selected these diseases.

4. I would like the authors to elaborate on the sensitivity analyses and the results in the discussion section a bit more.

We added more description in the discussion section.
